# Detecting *Thrips parvispinus* (Thysanoptera: Thripidae) in retail plant stores in Southern California

**DOI:** 10.1093/jisesa/ieag105

**Published:** 2026-09-09

**Authors:** Eric G Middleton, Nayeli Lee

**Affiliations:** Agriculture and Natural Resources, Cooperative Extension, University of California, San Diego, CA, USA; Agriculture and Natural Resources, Cooperative Extension, University of California, San Diego, CA, USA

**Keywords:** thrips, horticulture, invasive species

## Abstract

*Thrips parvispinus* (Thysanoptera: Thripidae: Karny) is an invasive pest that harms peppers and ornamentals. It has not been reported as present in Southern California but has a history of establishing in regions prior to official detection. We surveyed retail garden stores in San Diego and Orange Counties to determine if *T. parvispinus* was present in California but undetected, and found it on *Capsicum*, *Dipladenia*, *Mandevilla*, and *Gardenia* species across a year-long period. Our work demonstrates that *T. parvispinus* can spread to new areas undetected on nursery stock and validates sampling retail stores as a low-effort early detection method that can be used by researchers and regulators to find *T. parvispinus* on nursery stock.

## Introduction

*Thrips parvispinus* is an invasive thrips species in much of the world that can cause significant damage to peppers and numerous ornamental plants. Originally from Southeast Asia, *T. parvispinus* has since established in parts of Asia, Australia, Africa, Europe, and North America (Pijnakker 2023, Manideep et al. 2025). In North America, *T. parvispinus* is established in Hawaii, Florida, and Ontario, and has been detected in Colorado, North Carolina, South Carolina, Georgia, and Texas (Soto-Adames 2020, Pijnakker 2023, Neupane et al. 2026). While *T. parvispinus* has been observed feeding on numerous plant species, severe damage occurs most frequently in a subset of them, including *Capsicum*, *Mandevilla*, *Dipladenia*, and *Gardenia* (Mound and Collins 2000, Hulagappa et al. 2022).

*Thrips parvispinus* feeding causes leaf distortions in *Capsicum* and *Gardenia*, and distortions and notching in *Mandevilla* and *Dipladenia*, along with scarring (Hulagappa et al. 2022, Thorat et al. 2022). This damage can stunt plant growth, reduce the aesthetic value of hosts, and sometimes kill plants outright, making *T. parvispinus* a serious threat to the production of affected species.

Despite the threat *T. parvispinus* poses, there is still ambiguity regarding where it has spread in North America. After *T. parvispinus* was first officially detected in Florida, researchers surveyed retail stores in the state and found it in 22 of 23 stores, including in areas where *T. parvispinus* wasn’t known to be present (Ahmed et al. 2025). This indicates *T. parvispinus* can be present in areas well ahead of official detection.

Southern California, and San Diego County in particular, is at risk of *T. parvispinus* entering the region and has a large nursery and floriculture industry that could be threatened by this pest (San Diego County Agriculture Weights and Measures 2024, Beucke 2025). The climate is mild in Southern California, producers grow many common hosts of *T. parvispinus*, and nurseries frequently import plants from regions where *T. parvispinus* is known to be established. While *T. parvispinus* is not known to be established in California, it has previously been intercepted entering the state on plant material (Beucke 2025). Given *T. parvispinus* is a quarantine risk and has been shown to be present in areas before official detection (Mound and Collins 2000, Ahmed et al. 2025), it is possible *T. parvispinus* is already present in Southern California, yet has not been officially detected.

To monitor for the presence of *T. parvispinus* in Southern California, we conducted a survey of retail garden stores similar to the one conducted by researchers in Florida (Ahmed et al. 2025). Our goal was to determine if *T. parvispinus* was already present undetected in the region and to validate sampling retail stores as a monitoring program.

## Materials and Methods

**Fig. 1.**
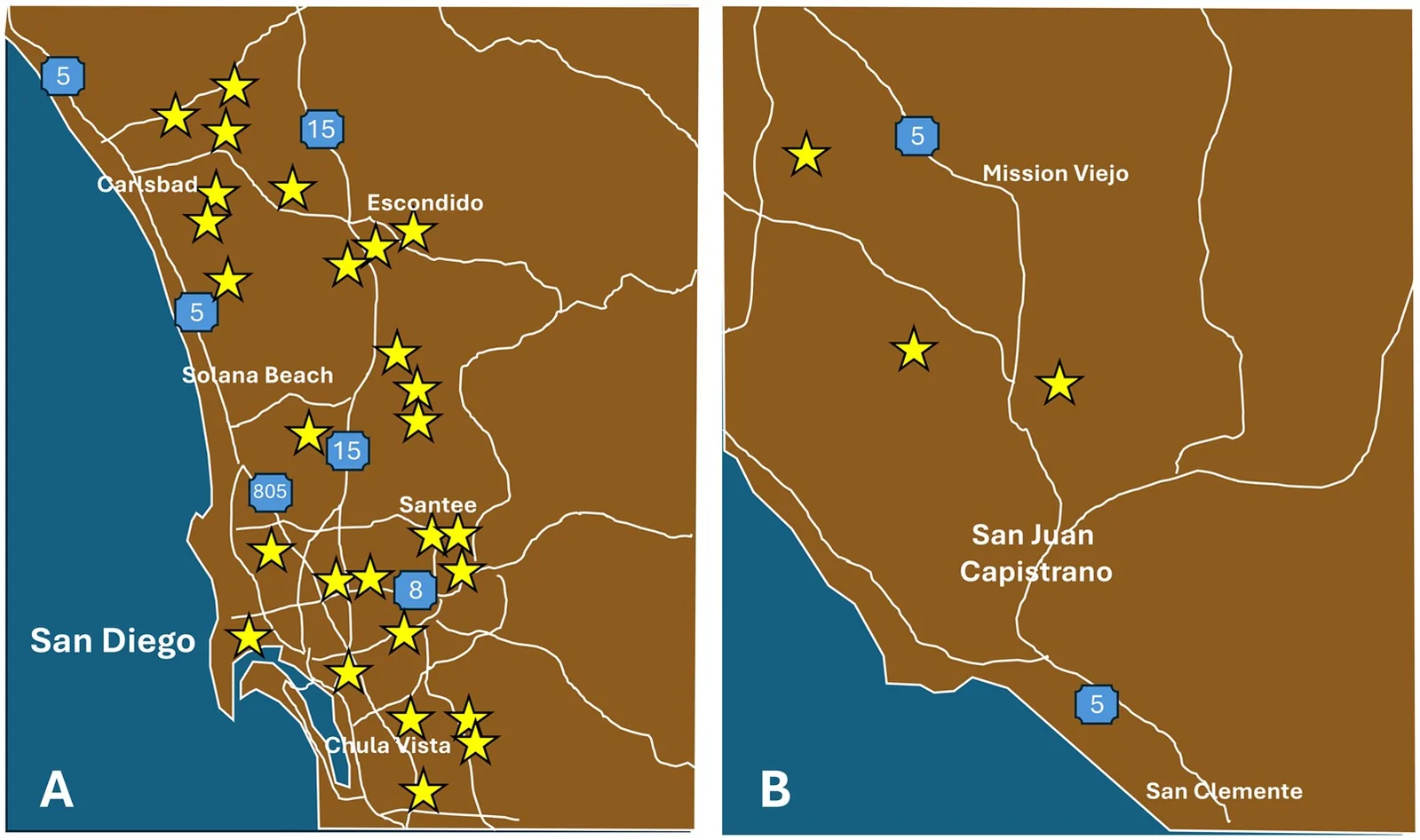
Maps of the retail garden stores (denoted by stars) we surveyed for thrips in A) San Diego County and B) Southern Orange County.

We sampled retail garden centers throughout San Diego County and into parts of southern Orange County (*n* = 27 and *n* = 3, respectively) (Fig. 1). We focused our sampling efforts on 4 different host plant types: *Capsicum spp.*, *Gardenia spp.*, *Dipladenia spp.*, and *Mandevilla spp*. When sampling at garden centers, we relied on in-store labels for plant identification, and all varieties and species within each of our 4 host plant groups were binned together for sampling. We also conducted a secondary survey of the same 4 host plants in publicly accessible areas in the landscape where host plants could be found.

Four rounds of sampling took place at garden centers. The first took place between 11 November 2024 and 12 December 2024; the second was between 20 January 2024 and 4 February 2024; the third was between 31 March 2025 and 18 May 2025; and the fourth was between 16 October 2025 and 14 January 2026. For host plants in the landscape, 1 round of sampling took place between 20 January 2025 and 4 February 2025.

We sampled thrips by tap-sampling plants. Twenty shoots and 20 flowers per host plant type were sampled per store unless not enough plants were available. Plant parts were held over 8 oz screw-cap plastic jars partially filled with ethanol and roughly tapped several times to dislodge any thrips present into the ethanol. If thrips were visible inside flowers but were not coming out with tap sampling, they were removed with a fine-tipped paintbrush and placed in the ethanol jar.

Thrips were identified using keys from Hoddle et al. (2012); and Jandricic et al. (2024); Initial *T. parvispinus* finds were verified by Michael Forthman with the California Department of Agriculture. *T. parvispinus* and western flower thrips (Thysanoptera: Thripidae: *Frankliniella occidentalis* Pergande) adults were identified to species, while all other adult thrips were classified as “Other Thrips.” Thrips larvae were not identified further and were classified as “Larvae” regardless of species.

We checked all host plants for damage indicative of *T. parvispinus* feeding on both new leaves and on flowers, if any were present. For leaves, damage on *Capsicum spp.* was crinkled and scarred leaves; *Dipladenia* and *Mandevilla* were notched, scarred, and distorted leaves; and *Gardenia* was yellowed, scarred, and distorted leaves. Damage on flowers for all host plants consisted of scarring and stippling. Shoots were considered damaged if at least 1 leaf per shoot showed clear signs of *T. parvispinus* damage.

## Results

**Fig. 2.**
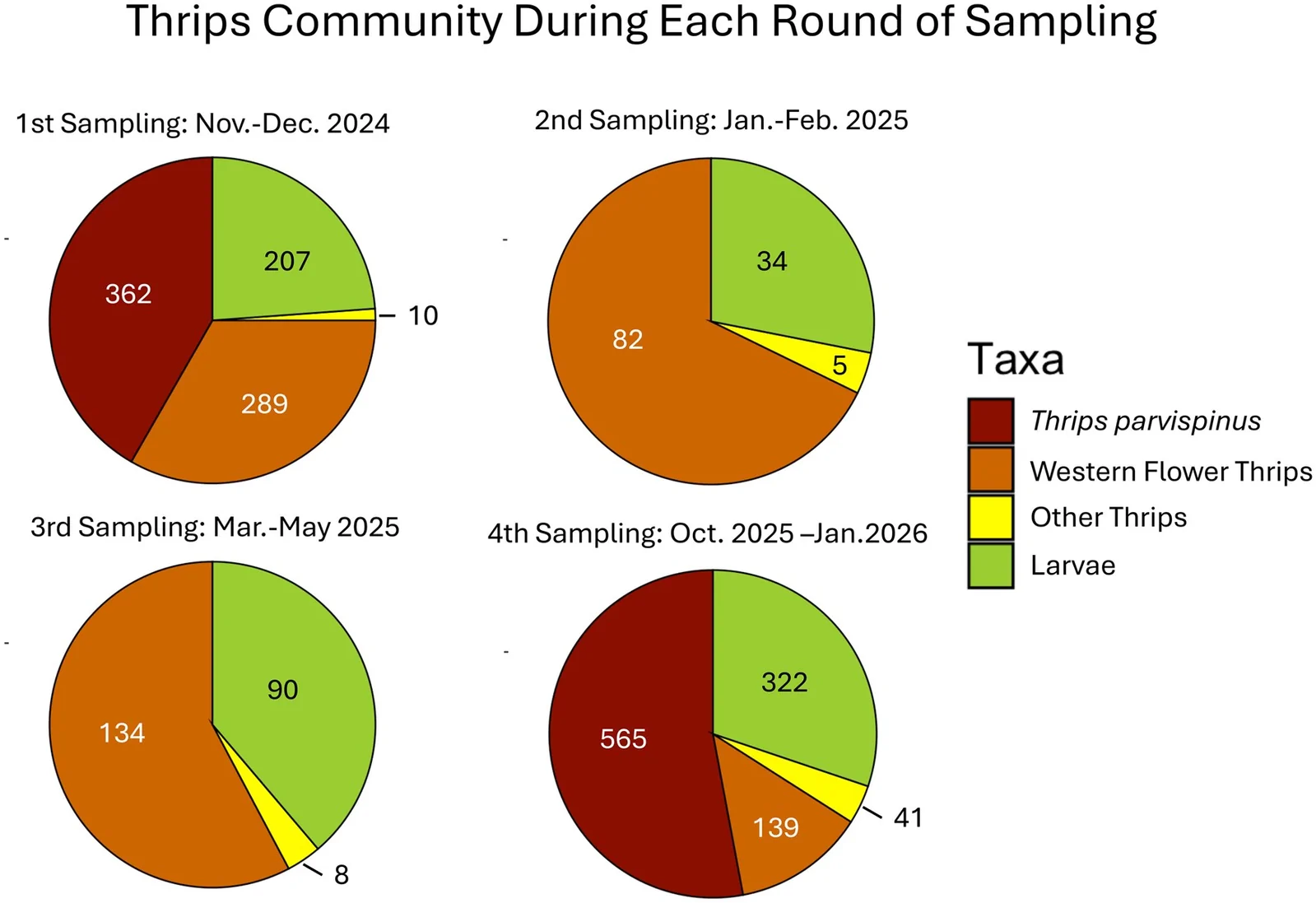
Pie graphs of the combined thrips community pooled across all host plants per round of sampling.

For the first round of sampling, we surveyed all 27 stores in San Diego County and 3 in Southern Orange County (Fig. 1). We found *T. parvispinus* and *T. parvispinus* feeding damage on all host plant types in 22 of the 27 San Diego County stores and in 2 of the 3 Orange County stores (Table 1). *T. parvispinus* made up the plurality of all the thrips we found in this survey (Fig. 2). In all cases, the stores where we didn’t find *T. parvispinus* didn’t have common host plants in stock. We observed multiple customers purchasing and leaving the store with host plants showing signs of *T. parvispinus* infestation.

**Table 1.** Number of flowers and shoots sampled, damaged flowers and shoots, and thrips found on host plants during the first round of sampling (11 November 2024 to 12 December 2024)

Host plant	Flowers sampled	Damaged flowers	*Thrips parvispinus*	Western flower thrips	Other thrips	Larvae
**Dipladenia**	533	62	157	74	0	37
**Mandevilla**	461	38	103	146	3	37
**Gardenia**	70	0	0	0	0	0
**Pepper**	0	0	0	0	0	0

Host plant	Shoots sampled	Damaged shoots	*Thrips parvispinus*	Western flower thrips	Other thrips	Larvae
**Dipladenia**	546	372	14	12	0	9
**Mandevilla**	505	434	11	29	1	31
**Gardenia**	765	554	62	5	2	69
**Pepper**	271	133	8	23	4	20

“Other Thrips” refers to any adult thrips that were not *T. parvispinus* or western flower thrips, and “Larvae” refers to all immature thrips regardless of species.

We reported our *T. parvispinus* finds to county regulatory agencies in San Diego and Orange County, and the majority of the infested plants were traced to a single grower in San Diego County. Approximately 600 infested Mandevilla plants at stores and 3,000 infested Gardenia plants at the nursery of origin were subsequently destroyed. After the destruction of the infested plants, we returned to stores to survey for *T. parvispinus* again.

During the second round of sampling, we surveyed 27 stores in San Diego County and 3 in Southern Orange County. We also surveyed 3 locations in the landscaping of colleges and public areas. Numerous other publicly accessible areas were scouted for host plants, and not enough were found to sample. We did not find *T. parvispinus* in any of the stores or landscape locations but did see symptoms consistent with *T. parvispinus* feeding damage on many hosts in stores (Table 2). Western flower thrips made up the majority of all thrips we found during this round of surveying (Fig. 2).

**Table 2.** Number of flowers and shoots sampled, damaged flowers and shoots, and thrips found on host plants during the second round of sampling (20 January 2024 to 4 February 2024)

Host plant	Flowers sampled	Damaged flowers	*Thrips parvispinus*	Western flower thrips	Other thrips	Larvae
**Dipladenia**	174	11	0	13	2	1
**Mandevilla**	147	10	0	9	0	1
**Gardenia**	0	0	0	0	0	0
**Pepper**	0	0	0	0	0	0

Host plant	Shoots sampled	Damaged shoots	*Thrips parvispinus*	Western flower thrips	Other thrips	Larvae
**Dipladenia**	511	227	0	7	3	0
**Mandevilla**	660	308	0	33	5	6
**Gardenia**	756	235	0	6	0	5
**Pepper**	442	163	0	15	16	4

“Other Thrips” refers to any adult thrips that were not *T. parvispinus* or western flower thrips, and “Larvae” refers to all immature thrips regardless of species.

For the third round of sampling, we surveyed all 27 stores in San Diego County. We did not find *T. parvispinus* in any of the stores during this round of sampling but again saw symptoms consistent with *T. parvispinus* feeding damage on many hosts (Table 3). Western flower thrips made up the majority of all thrips we found during this round of surveying (Fig. 2).

**Table 3.** Number of flowers and shoots sampled, damaged flowers and shoots, and thrips found on host plants during the third round of sampling (31 March 2025 to 18 May 2025)

Host plant	Flowers sampled	Damaged flowers	*Thrips parvispinus*	Western flower thrips	Other thrips	Larvae
**Dipladenia**	596	0	0	39	1	14
**Mandevilla**	598	0	0	27	2	33
**Gardenia**	0	0	0	0	0	0
**Pepper**	44	0	0	4	0	0

Host plant	Shoots sampled	Damaged shoots	*Thrips parvispinus*	Western flower thrips	Other thrips	Larvae
**Dipladenia**	615	143	0	8	0	5
**Mandevilla**	613	147	0	12	0	14
**Gardenia**	678	46	0	4	0	3
**Pepper**	578	194	0	40	5	21

“Other Thrips” refers to any adult thrips that were not *T. parvispinus* or western flower thrips, and “Larvae” refers to all immature thrips regardless of species.

During the fourth round of sampling, we surveyed 21 stores in San Diego County and found *T. parvispinus* and *T. parvispinus* feeding damage in all surveyed stores (Table 4). *T. parvispinus* made up the majority of all thrips we found during this round of surveying (Fig. 2). We again alerted San Diego and Orange County regulators and traced most of the infested plants back to the same nursery that had supplied infested plants in the first round of sampling. We visited the nursery supplying infested plants in Orange County on 19 November 2025, and sampled their supply of Mandevillas and Dipladenias. Most host plants had clear signs of *T. parvispinus* damage on their shoots. Of a total of 116 thrips collected at the nursery, 63 were *T. parvispinus*, 48 were western flower thrips, and 5 were thrips larvae.

**Table 4.** Number of flowers and shoots sampled, damaged flowers and shoots, and thrips found on host plants during the fourth round of sampling (16 October 2025 to 14 January 2026)

Host plant	Flowers sampled	Damaged Flowers	*Thrips parvispinus*	Western flower thrips	Other Thrips	Larvae
**Dipladenia**	238	3	196	53	0	27
**Mandevilla**	313	10	204	49	16	29
**Gardenia**	0	0	0	0	0	0
**Pepper**	42	0	54	13	1	87

Host Plant	Shoots Sampled	Damaged Shoots	*Thrips parvispinus*	Western flower thrips	Other Thrips	Larvae
**Dipladenia**	328	273	15	3	0	19
**Mandevilla**	370	236	5	2	0	18
**Gardenia**	466	451	84	1	6	110
**Pepper**	422	180	7	18	18	32

“Other Thrips” refers to any adult thrips that were not *T. parvispinus* or western flower thrips, and “Larvae” refers to all immature thrips regardless of species.

## Discussion

Our survey immediately detected *T. parvispinus* in Southern California stores where it was not known to be present, and our results are very similar to previous *T. parvispinus* surveys conducted in Florida stores (Ahmed et al. 2025). Our study further validates a survey of retail garden centers as a relatively low-effort and effective way to check for the presence of *T. parvispinus* in nursery stock and could help prevent the introduction of *T. parvispinus* into new areas. Sampling retail stores like this can catch pests that normal inspection methods fail to capture. During our study, for example, there were no instances of county agricultural offices in the region or the California Department of Food and Agriculture independently intercepting *T. parvispinus* on nursery stock, while our sampling demonstrated it was present and widespread in commercial stores. Researchers in other countries or states where *T. parvispinus* is not yet known to be present should consider this method as a potential early warning system. Based in part on the results of our survey, the California Department of Food and Agriculture listed *T. parvispinus* as an A-rated pest, indicating it poses a significant threat to California’s agriculture or environment (Beucke 2025).

Despite stores all over the region selling common host plants, we were unable to locate many of those same host plants in the landscape. Because of this, our inability to find *T. parvispinus* in the landscape shouldn’t be taken as evidence that it isn’t present. *T. parvispinus* survives and reproduces best at temperatures between 15 and 32 °C (Sridhar et al. 2025, Reyes-Arauz et al. 2026), which matches the Southern California climate for much of the year, although temperatures below 10 °C during winter months could lead to increased *T. parvispinus* mortality (Reyes-Arauz et al. 2026). Infested plants were present in stores and being sold for months on end, and we saw customers leaving stores with plants showing clear signs of *T. parvispinus* infestation. Taken together, it is possible that *T. parvispinus* could now be present in landscape plants in Southern California. Even if *T. parvispinus* does not persist well in the landscape, it could survive and reproduce in climate-controlled greenhouses or screenhouses of nurseries. *T. parvispinus* remains present in greenhouses in Ontario despite being unable to survive year-round in the landscape (Gleason et al. 2023).

Efforts on the part of regulators in San Diego County to eradicate *T. parvispinus* did seem to have an effect. During our second and third surveys, we found no *T. parvispinus* in any of the stores, indicating that the destruction of plants did eliminate or severely reduce *T. parvispinus* populations in stores for several months. However, *T. parvispinus* reappeared in stores a few months after the 3^rd^ round of sampling and reappeared in greater abundance than before. Whether an active *T. parvispinus* infestation persisted at the nursery supplying the plants to stores or whether their stock was reinfested from a new shipment from out-of-state is unclear. Future efforts to determine if *T. parvispinus* is present in the landscape or is being continuously reintroduced to nurseries should be carried out to better inform future regulatory and preventative action for this pest.

The future of *T. parvispinus* in Southern California remains uncertain. *T. parvispinus* has only been found in nursery stock so far and is not considered established. However, *T. parvispinus* may already be present in the landscape, and even if it isn’t, there is the ongoing potential for reintroduction to California. Overall, our results suggest that *T. parvispinus* may well be a continuous issue for growers in Southern California, and regulators, researchers, and industry members should remain vigilant for this pest in both nurseries and the landscape.
